# Isolation and characterization of the bioactive metabolites from the soil derived fungus *Trichoderma viride*

**DOI:** 10.1080/21501203.2017.1423126

**Published:** 2018-01-10

**Authors:** Nagwa E. Awad, Hanaa A. Kassem, Manal A. Hamed, Amal M. El-Feky, Mohamed A. A. Elnaggar, Khaled Mahmoud, Mohamed A. Ali

**Affiliations:** a Pharmacognosy Department, National Research Centre, Giza, Egypt; b Pharmacognosy Department, Faculty of Pharmacy, Cairo University, Giza, Egypt; c Therapeutic Chemistry Department, National Research Centre, Giza, Egypt; d Plant Pathology Department, National Research Centre, Giza, Egypt; e Center of Scientific Excellence for Influenza Viruses, National Research Centre, Dokki, Giza, Egypt

**Keywords:** *Trichoderma viride*, antioxidant, antimicrobial, anticancer, antiviral

## Abstract

The aim of the present study was to evaluate different biological activities of *Trichoderma viride* fungus (Family Hypocreaceae). *Trichoderma viride* isolated for the first time from the cucumber soil (rhizosphere). It was tested as antimicrobial, antioxidant and anticancer agent. *Trichoderma viride* from the cucumber soil (rhizosphere) caused inhibition of the mycelial growth of *Fusarium solani, Rhizoctonia solani* and *Sclerotium rolfsii*. Also, the alcoholic extract of the fungal mycelia proved a potent antibacterial activity against *Bacillus subtilis, Escherichia* coli and *Pseudomonas fluorescens*. In addition, it exhibited a significant antifungal activity against *Candida albicans, Fusarium solani, Fusarium oxysporium, Rhizoctonia solani* and *Pythium ultimum* at 100 µg/disc. Study of the antimicrobial and antioxidant activities of the volatile constituents had been done. The *in vitro* antioxidant, anticancer and antiviral activities of the isolated proteins, and carbohydrates were determined. Furthermore, the volatile constituents were isolated from fresh mycelia of *Trichoderma viride* and subjected to GC/MS analysis. Total protein (10%), carbohydrate (19.57%), steroidal (13.95%) and triterpenoidal content (38.34%) were determined in the alcoholic extract of *Trichoderma viride* mycelia. In conclusion, this fungus showed antioxidant, anticancer, antiviral and antibacterial effects. Further studies must be done to identify the molecules responsible for its effect and to consider its application in the pharmacological and medicinal purposes.

## 1. Introduction

Fungus is the second largest kingdom after insects (Baron 1996). Fungi occur in Antarctic ice, tropical and temperate regions, surface of mountain rocks and seawater (Feofilova 2001). They represent an enormous source for natural products with diverse chemical structures and activities (Hawksworth 1991). Fungi produce a vast range of secondary metabolites and they are known for their capacity to secrete high levels of enzymes, antibiotics, vitamins, polysaccharides and organic acids (Meyer 2008).

*Trichoderma viride* is well known for its antagonistic ability towards plant pathogenic fungi and they have received considerable attention as biocontrol agent of soil-borne plant pathogens (Mannina and Segrc 1997). The production of secondary metabolites by *Trichoderma* strains also shows great variety and application potential in the medicinal field. *Trichoderma viride* seems to be an inexhaustible source of antibiotics by transformation of the acetaldehydes gliotoxin and viridin (Dennis and Webster 1971), to alpha-pyrones (Keszler et al. 2000).

The aim of this work was to evaluate *Trichoderma viride* fungus as antimicrobial and *in vitro* antioxidant agent. The study also had been extended to the isolation and identification of volatile constituents, proteins and carbohydrates. In addition, the determination of their biological activities such as anticancer and antiviral activities had been evaluated.

## 2. Materials and methods

### 2.1. Fungal isolation

*Trichoderma viride* (Family Hypocreaceae) was isolated from the cucumber soil (rhizosphere), Omar Makram Farm (Beheara Governorate, Egypt) during autumn and winter growing season of 2012/2013. The isolate was incubated on potato dextrose agar (PDA; Becton and Dickinson Co., MA, USA) for 2 weeks at 25 ± 3°C. Isolated fungus was identified at the Plant Pathology Department, National Research Centre, Egypt, and confirmed by Fungal Taxonomy Department, Plant Pathology Research Institute, Agricultural Research Centre, Giza, Egypt, according to the morphological and culture characters using the methods described by Barnett and Hunter (1972) and Ramirez (1982).

### 2.2. Production and extraction of *Trichoderma viride*

Erlenmayer flasks (2 L) containing 400 ml potato dextrose broth were autoclaved at 121°C for 15 min and then inoculated with mycelium plugs from a 5-day-old culture of *Trichoderma viride* on PDA. The flasks were incubated for 2 weeks at 25°C, the air-dried powder (in shade) of the mycelia of *Trichoderma viride* was extracted with 70% ethyl alcohol.

### 2.3. Investigation of the volatile constituents

Two hundred grams of fresh mycelia of *Trichoderma viride* have been subjected to hydro-distillation for 3 h in a modified Likens and Nickerson apparatus (Macleod and Cave 1975) which allowed the distillation and simultaneous extraction of the volatile constituents in an organic solvent (n-pentane). The n-pentane layer was collected, dried over anhydrous sodium sulphate. The pentane layer was evaporated under reduced pressure, and stored in a refrigerator at 4°C for bioactivity and chemical analysis. The volatile oil constituents were subjected to GC/MS analysis. Identification of the components had been performed by comparing the retention times and mass spectra with those of the available database libraries [Wiley (Wiley Int.) USA and NIST (Nat. Inst. St. Technol., USA)] and/or published data (Adams 1989). Quantitative determination was carried out based on peak area integration.

### 2.4. Determination of total protein content

Determination of total protein content was carried out in the crude 70% ethyl alcohol extract of *Trichoderma viride* by micro-Kjeldahl’s method using Markham distillation apparatus according to Pearson (1970) through determination of nitrogen content. The Kjeldahl’s method is based on the principle of digestion of the substance with concentrated sulphuric acid; a process in which the nitrogen is converted into ammonia.

Isolation of proteins from the alcoholic extract was performed according to El-Gengaihi et al. (1996), where 5 g extract was dissolved in 20 ml water, 45 ml ethanol and 5 ml concentrated sulphuric acid. It left for 20 min at 27°C and 50 ml water and 250 ml absolute ethanol were added then filtered. The PH was adjusted to 3 by 28% ammonium hydroxide. Acetone was added till precipitate was formed and precipitated by centrifugation at 3000 r.p.m then collected, weighted and dried. The HPLC analysis of amino acids was performed using a model Eppendorf-Germany LC 3000 amino acid analyser (Widner and Eggum 1966).

### 2.5. Quantitative estimation of total carbohydrate content

Total carbohydrate content of the crude (70% alcoholic) extract of *Trichoderma viride* mycelia was estimated as glucose by phenol sulphuric acid method according to Dubois et al. (1956).

### 2.6. Isolation of mucilage

Mucilage was isolated from the crude extract of *Trichoderma viride* according to Laidlow and Percival (1950), where 5 g of the crude extract was dissolved in least amount of acidified water at pH 4 using concentrated hydrochloric acid. Four volumes of absolute ethanol were added dropwise till complete precipitation occurred. The precipitate was separated by centrifugation, washed several times with ethanol and then dried by freeze dryer to obtain a crude polysaccharide. HPLC analysis of polysaccharide hydrolysate was performed.

### 2.7. Quantitative estimation of total steroidal and terpenoidal content

Spectrophotometric method was based on measuring the intensity of the colour developed when sterols and terpenes react with Lieberman–Burchard reagent and the percentage was calculated as β-sitosterol (sterols) and β-amyrin (terpenes) according to SwiftMary (1984).

### 2.8. Determination of the antifungal activity

Biculture test was done following the methods of Soytong and Quimio (1989). A virulent isolate of *T. viride* was used in biculture test with different antagonistic fungi. An agar disc taken from the edge of radial growth of *F. solani, R. solani*, or *S. rolfsii* separately in PDA plate was obtained using a sterile cork borer and placed in one side of a PDA plate about 2.0 cm from the centre. An agar disc of *T. viride*, the antagonistic fungus was placed on the other side of the plate. For control treatment, the agar plug of only pathogen was placed on PDA plates. The biculture plates were incubated at room temperature until colony of control grew to full plate. At this point, colony diameter was measured using ruler. Zone diameter was measured to the nearest whole millimetre at the point wherein there was a prominent reduction of 80% growth.

Percentage of the growth inhibition was calculated using the following formul:

% inhibition = *A* − *B*/*A* × 100

where: *A* = colony diameter of pathogen in control, *B* = colony diameter in biculture.

### 2.9. Strains of tested microorganisms

The antimicrobial activity of the crude mycelia extract of *Trichoderma viride* was performed using antibiotic assay method according to Thabrew et al. (1997). The antibacterial activity was tested against *Bacillus subtilis* (Gram-positive bacteria), *Escherichia coli* and *Pseudomonas fluorescens* (Gram-negative bacteria) on nutrient agar medium. The antifungal activity was tested against the yeast (*Candida albicans*) and the phytopathogenic fungi (*Fusarium solani, Fusariumoxysporium, Rhizoctonia solani* and *Pythiumultimum*) using Sabouraud dextrose agar medium.

### 2.10. Determination of minimum inhibitory concentration

The minimum inhibitory concentration (MIC) of the crude mycelia extract of *Trichoderma viride* was determined by antibiotic assay method. Nutrient agar media was prepared and sterilised, then distributed in sterile petri dishes, each of 12 cm diameters. Each suspension of the test organisms was separately inoculated into the surface of a number of petri dishes. Each antibiotic assay disc (6 mm) was loaded with 50, 100, 200, 300 and 400 μg/disc of the crude mycelia extract of *Trichoderma viride*in dimethyl sulfoxide and firmly applied to the surface of the inoculated agar plates. The plates were observed for the growth of microorganisms. The lowest concentration of the extract inhibit the growth of the given bacteria/fungi was determined and considered as the MIC. The zone diameter is measured to the nearest whole millimetre at the point wherein there is a prominent reduction of 80% growth. Percentage of inhibition = *A* − *B*/*A* × 100, where *A* = colony diameter of control, *B* = colony diameter tested.

### 2.11. Antimicrobial activity of the alcoholic mycelia extract

The antimicrobial activity of the alcoholic mycelia extract was done using the disc diffusion method (Gnanamanickam and Mansfield 1981).The alcoholic extract at 100 μg/disc was screened *in vitro* for their antimicrobial activity against different pathogens (bacteria and fungi). Ampicillin (100 μg/disc) standard antibacterial and fluconazole (100 μg/disc) standard antifungal were used as reference drugs. This assay was replicated three times. All the experiments were done under aseptic conditions. Bacterial plates were incubated at 30°C for 24 h, while fungal plates were incubated at 28°C for 48 h. The diameter of the inhibition zone was recorded for each replicate and the average diameter was calculated.

### 2.12. Evaluation of antimicrobial activity of the volatile constituents

The volatile constituents isolated from the fresh fungus, *Trichoderma viride*, were subjected to evaluation of the antimicrobial activity using the disc diffusion method according to Thabrew et al. (1997). The antibacterial activity was tested against *Bacillus subtilis* (Gram-positive bacteria) and *Escherichia coli* (Gram-negative bacteria) on nutrient agar medium. The antifungal activity was tested against the yeast *Candida albicans* and the phytopathogenic fungi (*Fusarium solani* and *Rhizoctonia solani*) using Sabouraud dextrose agar medium.

### 2.13. Antioxidant activity of the isolated volatile constituents, proteins and carbohydrates

Volatile constituents, proteins and carbohydrates isolated from the fungus *Trichoderma viride* were *in vitro* evaluated as antioxidant agents. The antioxidant activities of serial concentrations of the proteins and carbohydrates (10, 50, 100 μg) were estimated by the method of Chen et al. (1999) using 2,2-diphenyl-1-picrylhydrazyl (DPPH^-^)-free radical. The decrease in optical density of DPPH^-^ was calculated in relation to control as follows:

% inhibition percentages = (control − sample/control) × 100

### 2.14. Evaluation of cytotoxicity of the isolated proteins and carbohydrates

Cytotoxicity study (*in vitro* bioassay on human hepatocellular carcinoma cell line; HepG2) of the isolated proteins and carbohydrates was determined by the Bioassay-Cell Culture Laboratory, National Research Centre, Giza, Egypt. Cell viability was assessed by the mitochondrial dependent reduction of yellow MTT (3-(4, 5-dimethylthiazol-2-yl)-2, 5-diphenyl tetrazolium bromide) to purple formazan according to Mosmann (1983). A positive control (adrinamycin) was used as a known cytotoxic natural agent (Thabrew et al. 1997). The experiment was performed in a sterile area using a laminar flow cabinet biosafety class II level (Baker, SG403INT and Sanford, ME, USA). The percentage of change in viability was calculated according to the following formula:

[(1 − (reading of extract))/(reading of negative control)] × 100.

A probit analysis was carried for IC50 and IC90 determination using SPSS 11 program.

### 2.15. Antiviral activity of the isolated proteins and carbohydrates

The assay was carried out according to the method of Hayden et al. (1980) in a six-well plate where MDCKM cells (10^5^ cells/ml) were cultivated for 24 h at 37°C. A/CHICKEN/QALUBIA/1/2006 (H5N1) virus was diluted to give 10^4^PFU/well and mixed with the concentration of the tested compounds; 1 µg/ml of L-1-(tosyl-amido-2-phenyl) ethyl chloromethyl ketone and incubated for 1 h at 37°C before being added to the cells. Growth medium was removed from the cell culture plates and virus-Cpd or virus-extract and Virus-Zanamivir mixtures were inoculated (100 µl/well). After 1 h contact time for virus adsorption, 3 ml of DMEM supplemented with 2% agarose was added onto the cell monolayer, plates were left to solidify and incubated at 37°C till formation of viral plaques (3–4 days). Formalin (10%) was added for 2 h and then plates were stained with 0.1% crystal violet in distilled water. Control wells were included where untreated virus was incubated with MDCK cells and finally plaques were counted and percentage reduction in plaque formation in comparison to control wells was recorded as follows:

% inhibition = viral count (untreated) – viral count (treated)/viral count (untreated) × 100.

### 2.16. Statistical analysis

Statistical analysis was carried out by independent student *t*-test using SPSS (Statistical Package for the Social Science; SPSS Inc., Chicago, IL, USA) Computer Program. Significance difference between groups was at *p* < 0.05.

## 3. Results

### 3.1. Fungal identification

**Figure 1.**
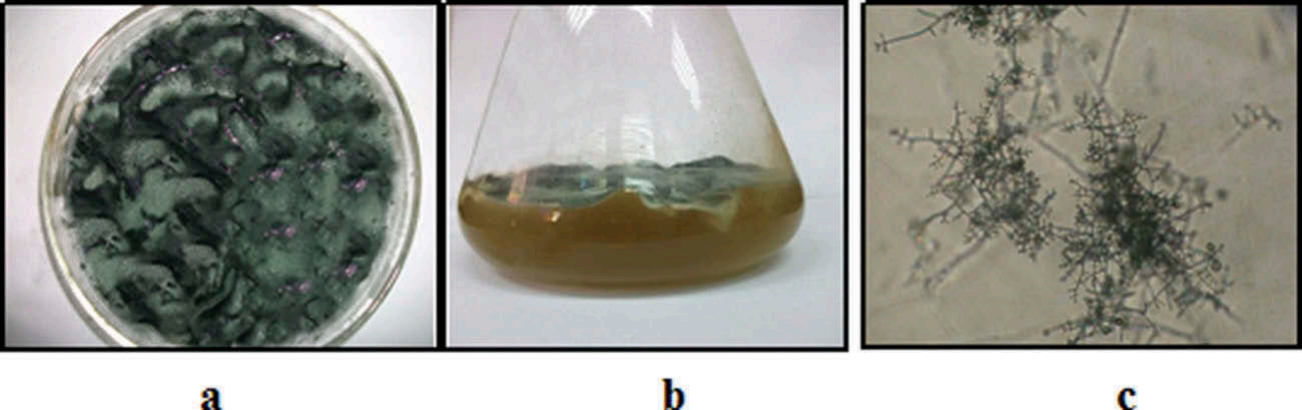
Petri dish (**a)**, flask (**b)** and microscopic features **(c)** of *Trichoderma viride.*

The isolate was identified as *Trichoderma viride* based on the criteria of Kirk et al. (2011). The colony was woolly and become compact in time. Colonies of *Trichoderma* grow rapidly and mature in 5 days. At 25°C and on PDA, the colonies were woolly and become compact in time. From the front, the colour is white. As the conidia were formed, scattered bluish green or yellowish green patches become visible (Figure 1). These patches may sometimes form concentric rings (St-Germain and Summerbell 1996).

The microscopic examination of *T. viride* revealed that the hyphae were septate, and conidiophores, phialides and the conidia were observed. Conidiophores were hyaline, branched and may occasionally display a pyramidal arrangement. Phialides were hyaline, flask-shaped and inflated at the base. They were attached to the conidiophores at right angles. The phialides may be solitary or arranged in clusters. Conidia (3 µm in diameter, average) were one-celled and round or ellipsoidal in shape. They were smooth-walled or rough-walled and grouped in sticky heads at the tips of the phialides. The colour of the conidia was mostly green (Sutton et al. 1998; Lieckfeldt et al. 1999).

The air-dried powder of the mycelia of *Trichoderma viride* was extracted with 70% ethyl alcohol, the yield was 75% (wt/wt % dried mycelia).

### 3.2. Volatile constituents

The yield of the volatile constituents was 0.19% of fresh mycelia. GC/MS analysis identified 30 compounds which represent 78.25% of the total volatile compounds. The volatile constituents were composed of hydrocarbons 3.61%, alcohols 18.76%, aldahyde 3.95%, ketones 2.47%, esters 3.56%, monoterpenes 11.83% and sesquiterpenes 34.07%. Cyclooctanol, caryophyllene oxide and α-bisabolol (8.48%, 5.12% and 5.04%, respectively) were represented as the major volatile constituents in *Trichoderma viride* mycelia as shown in Table 1.

**Table 1.** GC/MS analysis of the volatile constituents from the mycelia of *Trichoderma viride.*

Peak No.	Rt	Mol. formula	Mol. wt.	b.p.	Compound	%	Structure
1	3.98	C_8_H_16_O	128	57	Cyclooctanol	8.48	
2	4.59	C_6_H_10_O_3_	130	41	6,8-Dioxabicyclo octan-4-ol	2.45	
3	5.11	C_10_H_14_	134	119	Cymol	3.66	
4	6.19	C_9_H_10_O	134	134	Chavicol	1.20	
5	7.39	C_10_H_16_	136	68	Limonene	2.70	
6	8.65	C_9_H_16_O	140	81	6-Methyl-bicyclooctan-7-ol	1.66	
7	9.43	C_10_H_22_	142	43	n-Decane	1.32	
8	10.21	C_10_H_18_O	154	43	1,8-Cineole	1.48	
9	11.38	C_10_H_20_O_2_	172	43	1,2-Dihydro-8-hydroxylinalool	1.33	
10	15.26	C_10_H_12_O	148	148	Anethole	1.43	
11	16.44	C_10_H_16_O	152	81	2,4-Decadienal	2.50	
12	17.61	C_11_H_16_O	164	41	Cis-jasmone	2.47	
13	21.22	C_10_H_16_O_2_	168	43	Ascaridole	1.23	
14	21.78	C_12_H_20_O	180	81	2,4-Dodecadienal	1.45	
15	22.86	C_12_H_24_O	184	71	1-Methyl cycloundecanol	1.29	
16	24.66	C_12_H_24_O_2_	200	55	1,2-Cyclododecanediol	1.46	
17	25.91	C_15_H_24_	204	121	α-Humulene	2.45	
18	26.86	C_15_H_24_	204	41	Elemene	3.30	
19	27.11	C_15_H_24_	204	161	α-Copaene	2.23	
20	34.12	C_15_H_24_O	220	41	Caryophyllene oxide	5.12	
21	34.33	C_15_H_24_O	220	43	Ledene oxide	2.22	
22	34.58	C_15_H_26_O	222	82	α-Bisabolol	5.04	
23	35.04	C_15_H_26_O	222	161	Guaiol	1.82	
24	36.82	C_15_H_26_O	222	81	6-Epi-shyobunol	2.22	
25	37.49	C_15_H_26_O	222	59	Elemol	2.11	
26	38.69	C_15_H_26_O	222	119	α-Acorenol	3.81	
27	40.60	C_15_H_26_O_2_	238	43	Cedrane-8,13-diol	3.75	
28	41.76	C_16_H_30_O_2_	254	55	1,215,16-Diepoxyhexadecane	2.29	
29	46.62	C_21_H_34_O_2_	318	41	Methyl arachidonate	3.56	
30	50.29	C_37_H_76_O	536	43	1-Heptatriacotanol	2.22	
Total identified compounds						78.25%	

### 3.3. Total protein content

The crude extract of the fungus *Trichoderma viride* gave positive tests for proteins. The estimation of protein in the crude extract revealed that it contains 10% protein (wt/wt) of the dried crude extract.

Isolation of the proteins gave a percentage of 8.5% (wt/wt) of the dried crude extract. HPLC analysis led to identification of seven essential amino acids represented 38.39% (wt/wt) of the total amino acids and nine non-essential amino acids represented 61.61% (wt/wt) of the total amino acids. Lysine and methionine were detected as major essential amino acids and represented 7.23% and 11.88% (wt/wt), respectively,of the total amino acids content. However, glutamic acid was found as a major non-essential amino acid and represented 13.92% (wt/wt) of the total amino acid content as illustrated in Table 2.

**Table 2.** Total amino acids of the crude extract of *Trichoderma viride* mycelia.

Amino acids	RT (min.)	Relative percentage of total amino acids
Threonine	13.08	3.34
Valine	30.73	3.88
Methionine	35.52	11.88
Isoleucine	38.15	2.88
Leucine	39.90	3.26
Phenylalanine	44.78	5.92
Lysine	53.20	7.23
Total essential amino acids		38.39
Aspartic	10.13	7.83
Serine	13.93	5.26
Glutamic acid	15.52	13.92
Glycine	22.52	2.33
Proline	22.43	4.29
Alanine	24.13	7.57
Tyrosine	42.90	6.96
Histidine	50.20	7.48
Arginine	61.48	5.97
Total nonessential amino acids		61.61
Total identified amino acids		100

### 3.4. Total carbohydrates

The crude extract of *T. viride* mycelia gave positive test for carbohydrates. The estimation of the total carbohydrate content revealed that the alcoholic extract of *T. viride* mycelia contained 19.57% (wt/wt) of the dried extract.

### 3.5. Mucilage contents

Isolation of the mucilage gave a percentage of 17.5 (wt/wt) of the dried crude extract. HPLC analysis of the mucilage hydrolysate revealed the identification of five free sugars which represented 85.791% (wt/wt) of the total mucilage hydrolyzate. Glucose was presented as a major sugar (29.32% (wt/wt) of the total mucilage hydrolysate), followed by galacturonic acid, galactose, fructose and arabinose (Table 3).

**Table 3.** HPLC analysis of mucilage hydrolysateof the 70% ethyl alcohol extract of *Trichoderma viride* mycelia.

Authentic sugars	R.T. (min.)	Relative percentage (wt/wt%) of the total mucilage hydrolysate
Galacturonic acid	6.601	16.901
Glucose	7.712	29.320
Galactose	8.033	8.447
Fructose	9.533	21.583
Arabinose	9.566	9.540
Total identified sugars	85.791	

### 3.6. Total steroidal and terpenoidal contents

The absorbance of tested extract was 2.549 corresponding to 0.0005 g in 2 ml of the colour reagent. So, the total sterols and terpenes in the crude extract of *T. viride* mycelia were 13.95% and 38.34% (wt/wt), respectively, of the dried extract calculated as β-sitosterol and β-amyrin.

### 3.7. Antifungal activity

In the control plate, the pathogenic fungi grow faster and significantly formed larger colony diameter with a mean of 8.97 cm, while those in the biculture plate produced smaller colony with a mean diameter of 6.30 cm for *Fusarium solani*, 7.60 cm for *Rhizoctonia solani* and 7.20 cm for *Sclerotium rolfsii*.

**Figure 2.**
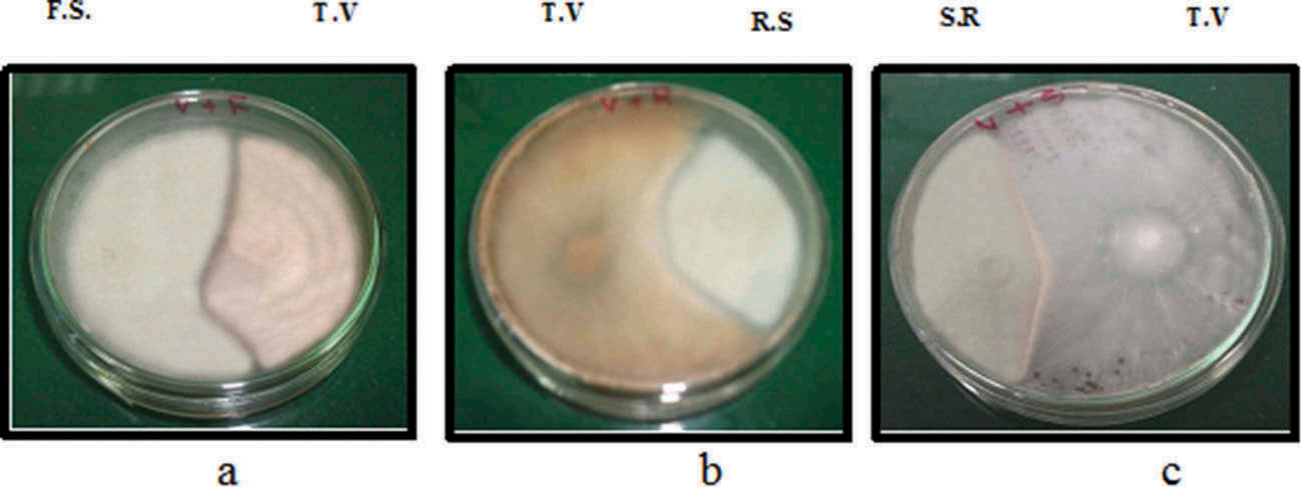
Biculture antagonistic test between *Trichoderma viride* (T.V.) and *Fusarium solani* (F.S.) (a), *Rhizoctonia solani* (R.S.) (b) and *Sclerotium rolfsii* (S.R.) (c).

*Trichoderma viride*, the fungal antagonist, caused 29.76, 15.27 and 19.73% inhibition of mycelial growth of *Fusarium solani, Rhizoctonia solani* and *Sclerotium rolfsii*, respectively, as illustrated in Table 4 and Figure 2.

**Table 4.** The colony diameters and the percentages of inhibition of *Trichoderma viride* against different pathogenic fungi.

Treatment	Colony diameter (mm) mean ±S.D	Inhibition %
Pathogens alone	8.97 ± 0.21	**–**
*Fusarium solani* in Biculture	6.30٭± 0.75	29.76
*Rhizoctonia solani* in Biculture	7.60٭±0.55	15.27
*Sclerotium rolfsii* in Biculture	7.20٭± 0.74	19.73

Data are mean± SD of triplicate reading of the colony diameter. ٭Significantly different from the control at *p ≤* 0.05 using one-way analysis of variance test.

### 3.8. Antimicrobial activity of the crude mycelia extract

The crude extract of *Trichoderma viride* was tested in 50, 100, 200, 300 and 400 µg/disc. Concentration of 50 µg/disc showed no significant inhibition zone on the tested pathogenic organisms (bacteria/fungi), while 100 µg/disc inhibited the growth of the given bacteria/fungi. The zone of inhibition was determined and this concentration was considered as the MIC. Concentration of 100 µg/disc of the alcoholic extract giving remarkable inhibition zone on *Bacillus subtilis, Escherichia coli* and *Pseudomonas fluorescens*. Maximum antifungal effect against *Candida albicans, Rhizoctonia solani, Pythium ultimum, Fusarium solani* and *Fusarium oxysporium* were recorded, the zones of the inhibition were increased as the concentration of the extract increase (Table 5).

**Table 5.** Antimicrobial effects of the alcoholic mycelia extract of *Trichoderma viride.*

	Diameter of inhibition zone (mm) mean ± SD				
Treatment	Concentration (µg/disc)	100 µg/disc	200 µg/disc	300 µg/disc	400 µg/disc
Bacteria	*B. subtilis*	12.06 ± 0.115^a^	13.10 ± 0.173^a^	13.66 ± 0.577^a^	14.33 ± 1.050^a^
	*E. coli*	12.06 ± 0.115^a^	12.16 ± 0.152^a^	13.13 ± 0.230^a^	15.13 ± 0.230^a^
	*P. fluorescens*	12.16 ± 0.288^a^	12.33 ± 0.577^a^	13.66 ± 0.577^a^	15.00 ± 1.000^a^
Fungi	*C. albicans*	12.33 ± 0.577	12.93 ± 0.404	13.43 ± 0.513^a^	14.66 ± 0.577^a^
	*F. solani*	11.93 ± 0.378^a^	12.83 ± 0.568	14.13 ± 0.907^a^	15.30 ± 0.264^a^
	*F. oxysporium*	12.33 ± 0.577	14.26 ± 0.461^a^	14.13 ± 0.230^a^	15.33 ± 1.050^a^
	*R. solani*	11.66 ± 0.577	12.26 ± 0.461^a^	15.33 ± 0.577^a^	16.16 ± 0.208^a^
	*P. ultimum*	10.00 ± 1.00^a^	11.66 ± 0.577^a^	12.26 ± 0.251^a^	14.33 ± 0.321^a^

Data are mean± SD of triplicate reading of the inhibition zone diameter. ^a^Significantly different from the control at *p ≤* 0.05 using one-way analysis of variance test.

### 3.9. Antimicrobial activity of the volatile constituents

Volatile constituents isolated from the fresh fungus *Trichoderma viride* was evaluated as antimicrobial agent (antibacterial and antifungal). The diameter of inhibition zone (mm) for each pathogen was determined exactly and the mean of the triplicates was calculated. The volatile constituents under study showed remarkable antibacterial and antifungal effects at concentration of 100 µl/disc as shown in Table 6.

**Table 6.** Antimicrobial effects of the volatile constituents isolated from the fresh fungus *Trichoderma viride.*

Treatment		Diameter of inhibition zone (mm) mean ±SD
Bacteria	*B. subtilis*	13.16 ± 0.208
	*E. coli*	11.33 ± 0.577
Fungi	*C. albicans*	9.16 ± 0.763
	*F. solani*	13.53 ± 5.774
	*R. solani*	12.66 ± 0.577

Data are mean± SD of triplicate reading of the inhibition zone diameter.

### 3.10. Antioxidant activity of the isolated volatile constituents, proteins and carbohydrates

The antioxidant activity of the isolated volatiles, proteins and carbohydrates were determined as illustrated in Table 7. The isolated volatile constituents revealed high antioxidant effects by 29.62%, 63.12% and 70.37% at concentrations of 10, 50 and 100 µg, respectively. Moreover, proteins and carbohydrates recorded remarkable antioxidant effects by 3.70%, 14.81% and 33.00% for proteins and 3.00%, 18.00% and 23.00% for carbohydrates at concentrations of 10, 50 and 100 µg, respectively.

**Table 7.** Percentages of the antioxidant activity of the isolated volatile constituents, proteins and carbohydrates.

Inhibition percentages (%) at different concentrations			
Concentration Treatment	10 µg	50 µg	100 µg
Volatile constituents	29.62	63.12	70.37
Proteins	3.70	14.81	33.00
Carbohydrates	3.00	18.00	23.00

Data are inhibition percentages (%) of DPPH^-^-free radicals at different concentrations.

### 3.11. Cytotoxicity of the isolated proteins and carbohydrates

The isolated proteins and carbohydrates exhibited 42.6% and 16.7% killing of the HepG2 cell line, as compared to the standard reference natural drug adrinamycin.

### 3.12. Antiviral activity of the isolated proteins and carbohydrates

The antiviral activity of the isolated proteins and carbohydrates recorded 20% and 18%, respectively, at a concentration of 25 µg/µl (Table 8).

**Table 8.** Antiviral activity of the isolated proteins and carbohydrates.

Treatment	Initial viral count (X ± SE)	Viral count after treatment (X ± SE)	Inhibition %
Isolated proteins	4.000 ± 0.0066	3.240 ± 0.0057	20
Isolated carbohydrates	3.036 ± 0.0033	2.480 ± 0.0057	18

Each value (X ± SE) represents the mean of the viral count ± SE.

## 4. Discussion

*Trichoderma viride* is the most promising and effective biocontrol agent to control the plant diseases as well as increase the plant growth. It has the antagonising ability to control a wide range of microorganisms for more than seven decades ago (Weinding 1934), but its use under field conditions came much later (Chet et al. 1997). In this study, *Trichoderma viride* fungus has exerted an inhibitory activity against the mycelial growth of *Fusarium solani, Rhizoctonia solani* and *Sclerotium rolfsii*. So, it can be used clearly as a potential antagonist of various plant pathogens. The mechanism of mycoparasitism involves nutrient competition, hyperparasitism and antibiosis (Ajith and Lakshmidevi 2010). Recently, the role of extracellular enzymes has been well documented by several researchers such as proteolytic enzymes (Pozo et al. 2004; Kredics et al. 2005), β-1,3-glucanolytic system (Kubicek et al. 2001), β-1,6-glucanases (De La Cruz et al. 1995; De La Cruz and Llobell 1999), α-1,3-glucanases (Ait-Lahsen et al. 2001), chitinase (Zeilinger et al. 1999; Hoell et al. 2005) and proteases (Geremia et al. 1993), which are considered as key factors in the pathogen cell wall lysis during mycoparasitism (Brito-Vega et al. 2013).

Recently, *Trichoderma viride* fungus has an important role in the human health, where this fungus is capable of remediating the heavy metals, toxins such as cyanides and xenobiotics, and converts the anthracene to non-toxic naphthalene (Harman 2006). So, it can be used for bioremediation of pollutants. Moreover, the fungus *Trichoderma viride* produces several important secondary metabolites such as the viridian-analouge, trichosetin and peptaibiotics which were used as potential anticancer and antimicrobial drugs (Harman 2006).

The formation of the secondary metabolites is frequently limited to the external conditions, where the fungi employ unique biochemical pathway for synthesis of the bioactive metabolites from few precursors (Demain and Fang 2000). Fungal volatile constituents are synthesised from various precursors such as acetates, amino acids, fatty acids and keto acids. Monoterpenes and sesquiterpenes are biosynthesized from acetyl COA and converted to the final structure of the compound by the action of terpene synthase (Sivasithamparam and Ghisalberti 1998). Various other groups of the volatile constituents such as the alcohols, aldehydes and ketones are presented and derived from the fatty acids. The volatile constituents can influence the physiological processes of the fungi including nitrification, nitrogen mineralization and participation in the developmental processes of the fungi. It acts as a defence system and plays an important role in the communication of the fungus *Trichoderma viride* with the other microorganisms, insects and plants (Harman 2006). These observations confirmed our results through isolation and identification of the volatile constituents from the fresh fungus, *Trichoderma viride* as well as the determination of the total proteins, carbohydrates, steroidal and terpenoidal contents.

The antifungal activities of the fungus *Trichoderma viride* recorded antifungal effect against *Fusarium solani, Rhizoctonia solani* and *Sclerotium rolfsii*. Also, the antimicrobial activities of the crude mycelia extract recorded remarkable antibacterial effect on *Bacillus subtilis, Escherichia coli* and *Pseudomonas fluorescens*. It also recorded maximum antifungal effect against *Candida albicans, Rhizoctonia solani, Pythium ultimum, Fusarium solani* and *Fusarium oxysporium*.

The volatile constituents isolated from the fresh *Trichoderma viride* mycelia revealed promising antifungal, antibacterial and high antioxidant effects. Also, the isolated proteins and carbohydrates recorded remarkable cytotoxic effect on HepG2 cell line, moderate antiviral activity on H5N1 virus and antioxidant effect by inhibition of DPPH^-^-free radical.

These observations are in line with the results of Gajera et al. (2016) who recorded antioxidant effect of *Trichoderma viride* against different pathogens. The anticancer activity of fungi has been verified to be connected with a variety of phytochemicals, such as polyphenols, flavonoids and catechins. Several recent studies have investigated the cytotoxicity of natural extracts against human cancer cell lines, where proliferation and viability of cancer cells were decreased after treatment with natural extracts. Cytotoxic activities of *Trichoderma* spp. against the liver cancer cell lines has not been studied enough. This is the first study that has used metabolites of the whole culture filtrate of *Trichoderma viride* as anticancer agents. Certain species of *Trichoderma* have been known as a productive producer of important secondary metabolites such as antibiotics, plant growth regulators and enzymes, which are mainly used to protect plants from pathogens (Respinis et al. 2010). The mechanism of action of *Trichoderma* as a biocontrol agent is a complex process mediated by the secretion of extracellular enzymes. Some enzymes produced by the *Trichoderma* species are known to have antitumor and antioxidant activity (Abd El-Rahman et al. 2014).

In conclusion, the cultural filtrates of *Trichoderma viride* are considered as the most promising and effective agents controlling wide range of microorganisms. Moreover, *Trichoderma viride* fungus proved to have various antimicrobial, antioxidant, anticancer and antiviral activities. Further studies are required to understand the mechanism(s) of action of these cultural filtrates on liver cancer cell line and against different pathogens. In addition, the study of *Trichoderma* spp. as a source of biologically active metabolites is especially significant and ensures interest on this subject for years to come.

## References

[CIT0001] Abd El-Rahman AA, ElShafei SMA, Ivanova EV, Fattakhova AN, Pankova AV, El-Shafei MA, El- El-Morsi MA, Alimova FK. 2014 Cytotoxicity of *Trichoderma spp*. cultural filtrate against human cervical and breast cancer cell lines. Asian Pac J Cancer Prev. 15:7229–7234. 25227819 10.7314/apjcp.2014.15.17.7229

[CIT0002] Adams P. 1989 Identification of essential oils by ion trap mass spectroscopy. New York: Academic Press, INC.

[CIT0003] Ait-Lahsen H, Soler A, Rey M, De La Cruz J, Monte E, Liobell A 2001 An antifungal exo-ᾳ-1,3-glucanase (AGN13.1) from the biocontrol fungus *Trichoderma harzianum*. Appl Environ Microbiol. 67:5833–5839. 11722942 10.1128/AEM.67.12.5833-5839.2001 PMC93379

[CIT0004] Ajith PS, Lakshmidevi N 2010 Effect of volatile and non-volatile compounds from *Trichoderma spp*. against *Colletotrichum capsici* incitant of anthracnose on bell peppers. Nat Sci. 8:265–269.

[CIT0005] Barnett HL, Hunter BB 1972 Illustrated genera of imperfect fungi. Minneapolis: Burgess Publ. com; p. 241.

[CIT0006] Baron S 1996 Introduction to Mycology. 4th ed. Galveston: University of Texas Medical Branch. 21413342

[CIT0007] Brito-Vega H, Espinosa-Victoria D, Salaya-Domínguez JM, Gómez-Méndez1 E 2013 The soil biota: importance in agroforestry and agricultural systems. Tropical and Subtropical Agroecosystems. 16:445–453.

[CIT0008] Chen Y, Wang M, Rosen RT, Ho CT 1999 2,2-Diphenyl-2-picrylhydrazyl radical-scavenging active components from *Polygonum multiflorum* Thunb. J Agric Food Chem. 47:2226–2228. 10794614 10.1021/jf990092f

[CIT0009] Chet I, Inbar J, Hadar I 1997 Fungal antagonists and mycoparasites In: Wicklow DT, Soderstorm B, eds.. Berlin: Springer, The Mycota IV: environmental and microbial relationships. p. 165–184.

[CIT0010] De La Cruz J, Llobell A 1999 Purification and properties of a basic endo-b-1,6-glucanase (BGN16.1) from the antagonistic fungus *Trichoderma harzianum*. Eur J Biochem. 265:145–151. 10491168 10.1046/j.1432-1327.1999.00698.x

[CIT0011] De La Cruz J, Pintor-Toro JA, Benitez T, Llobell A 1995 Purification and characterization of an endo-b-1,6-glucanase from *Trichoderma harzianum* that is related to its mycoparasitism. J Bacteriol. 177:1864–1871. 7896713 10.1128/jb.177.7.1864-1871.1995 PMC176818

[CIT0012] Demain AL, Fang A 2000 The natural function of secondary metabolites. Advanced in Biochemical engineering/Biochemistry. Heidelberg (Germany): Springer; p. 1–39. 10.1007/3-540-44964-7_1 11036689

[CIT0013] Dennis C, Webster J 1971 Antagonistic properties of species groups of *Trichoderma*: III. Hyphal interactions. Trans Br Mycol Soc. 57:363–369.

[CIT0014] Dubois M, Gilles KA, Hamilton JK, Rebers PA, Smith F 1956 Colorimetric method for determination of sugars and related substances. AnalChem. 28:350–356. 10.1038/168167a0 14875032

[CIT0015] El-Gengaihi S, Karawya M, Selim M, Motawe H, Ibrahim N 1996 Chemical and biological investigation of polypeptides of Monordica and Luffa Dpp. Fam. Cucurbitaceae. J Bull NRC. 21:269–276.

[CIT0016] Feofilova EP 2001 The kingdom fungi: heterogeneity of physiological and biochemical properties and relationships with plants, animals, and prokaryotes. Appl Biochemistry Microbiol. 37:124–137. 11357416

[CIT0017] Gajera HP, Katakpara ZA, Patel SV, Golakiya BA 2016 Antioxidant defense response induced by *Trichoderma viride* against *Aspergillus niger* Van Tieghem causing collar rot in groundnut (*Arachis hypogaea* L.). Microb Pathog. 91:26e–34. 26620080 10.1016/j.micpath.2015.11.010

[CIT0018] Geremia RA, Goldman GH, Jacobs D, Ardiles W, Vila SB, Van Montagu M, Herreraestrella A 1993 Molecular characterization of the proteinase-encoding gene, prb1, related to mycoparasitism by *Trichoderma harzianum*. Mol Microbiol. 8:603–613. 8326868 10.1111/j.1365-2958.1993.tb01604.x

[CIT0019] Gnanamanickam SS, Mansfield JW 1981 Selective toxicity of wyerone and other phytoalexins to gram-positive bacteria. Phytochemistry. 20:997–1000.

[CIT0020] Harman GE 2006 Overview of mechanisms and uses of *Trichoderma* spp. Phytopathology. 96:190–194. 18943924 10.1094/PHYTO-96-0190

[CIT0021] Hawksworth DL 1991 The fungal dimension of biodiversity: magnitude, significance, and conservation. Mycol Res. 95:641–655.

[CIT0022] Hayden FG, Cote KM, Douglas RG 1980 Plaque inhibition assay for drug susceptibilitytesting of influenza viruses. Antimicrob Agents Chemother. 17:865–870. 7396473 10.1128/aac.17.5.865 PMC283889

[CIT0023] Hoell IA, Klemsdal SS, Vaaje-Kolstad G, Horn SJ, Eijsink VGH 2005 Overexpression and characterization of a novel chitinase from *Trichoderma atroviride* strain P1. Biochim Biophys Acta. 1748:180–186. 15769595 10.1016/j.bbapap.2005.01.002

[CIT0024] Keszler A, Forgacs E, Kotal L, Vizcaino JA, Monte E, Garcia-Acha I 2000 Separation and identification of volatile components in the fermentation broth of *Trichoderma atroviride* by solidphase extraction and gas chromatography-mass spectroscopy. J Chromatograph Sci. 38:421–424. 10.1093/chromsci/38.10.421 11048777

[CIT0025] Kirk PM, Cannon PF, Minter DW, Stalpers JA 2011 Dictionary of the fungi. 10th ed. UK: Wallingford; p. 131.

[CIT0026] Kredics L, Zsuzsanna A, Szekeres A, Hatvani L, Manczinger L, Cs V, Erzsebet N 2005 Extracellular proteases of *Trichoderma* species-a review. Acta Microbiol Immunol Hung. 52:169–184. 16003937 10.1556/AMicr.52.2005.2.3

[CIT0027] Kubicek CP, Mach RL, Peterbauer CK, Lorito M 2001 *Trichoderma*: from genes to biocontrol. J Plant Pathol. 83:11–23.

[CIT0028] Laidlow M, Percival V 1950 The Chemistry of Gum and Mucilage. New York: Rembold Publishing Co.

[CIT0029] Lieckfeldt E, Samuels GJ, Nirenberg HI, Petrini O 1999 A morphological and molecular perspective of *Trichoderma viride*: is it one or two species. Appl Environ Microbiol. 65:2418–2428. 10347022 10.1128/aem.65.6.2418-2428.1999 PMC91357

[CIT0030] Macleod AJ, Cave SJJ 1975 Volatile flavour components of egg. Sci Food Agric. 26:351–358.

[CIT0031] Mannina L, Segrc AL 1997 A new fungal growth inhibitor from *Trichoderma viride*. Tetrahedron. 53:3135–3144.

[CIT0032] Meyer V 2008 Genetic engineering of filamentous fungi-Progress, obstacles and future trends. Biotechnol Adv. 26:177–185. 18201856 10.1016/j.biotechadv.2007.12.001

[CIT0033] Mosmann T 1983 Rapid colorimetric assays for cellular growth and survival: application to proliferation and cytotoxicity assays. J Immunol Methods. 65:55–63. 6606682 10.1016/0022-1759(83)90303-4

[CIT0034] Pearson D 1970 The chemical analysis of foods. 6th ed. London: Churchill LTD; p. 9.

[CIT0035] Pozo MJ, Baek JM, Garcia JM, Kenerley CM 2004 Functional analysis of tvsp1, a serine protease-encoding gene in the biocontrol agent *Trichoderma virens*. Fungal Genet Biol. 41:336–348. 14761794 10.1016/j.fgb.2003.11.002

[CIT0036] Ramirez C 1982 Manual and Atlas of the Penicilla. Amesterdam (New York, Oxford): Elserier Biomedical Press; p. 123.

[CIT0037] Respinis SD, Vogel G, Benagli C 2010 MALDI-TOF MS of Trichoderma: model system for the identification of microfungi. Mycol Prog. 9:79–100.

[CIT0038] Sivasithamparam K, Ghisalberti EL 1998 Secondary metabolism in *Trichoderma* and *Gliocladium. Trichoderma* and *Gliocladium*. Vol.1, Basic Biology, Taxonomy and Genetics. London: Taylor and Francis Ltd; p. 139–191.

[CIT0039] Soytong K, Quimio TH 1989 Antagonism of *Chaetomium globosum* to the rice blast pathogen. Pyricularia Oryzae Kasetsart J Nat Sci. 23:198–203.

[CIT0040] St-Germain G, Summerbell R 1996 Identifying filamentous fungi - a clinical laboratory handbook. 1st ed. Belmont (California): Star Publishing Company.

[CIT0041] Sutton DA, Fothergill AW, Rinaldi MG 1998 Guide to clinically significant fungi. 1st ed. Baltimore: Williams & Wilkins.

[CIT0042] SwiftMary L 1984 Analysis of molluscan sterols: colorimetric methods. Lipids. 19:625–630. 27520514 10.1007/BF02534722

[CIT0043] Thabrew MI, Hughes RD, McFarlane IG 1997 Screening of hepatoprotective plant components using a HepG2 cell cytotoxicity assay. J Pharm Pharmacol. 49:1132–1135. 9401951 10.1111/j.2042-7158.1997.tb06055.x

[CIT0044] Weinding R 1934 Studies on a lethal principle effective in the parasitic action of *Trichoderma lignorum* on *Rhizoctonia solani* and other soil fungi. Phytopath. 24:1153–1179.

[CIT0045] Widner K, Eggum OB 1966 Protein analysis. A description of the method used at the of animal physiology in Copenhagen. Acta Agricultura Scandinavi. 16:15.

[CIT0046] Zeilinger S, Galhaup C, Payer K, Woo SL, Mach RL, Fekete C, Lorito M, Kubicek CP 1999 Chitinase gene expression during mycoparasitic interaction of *Trichoderma harzianum* with its host. Fungal Genet Biol. 26:131–140. 10328983 10.1006/fgbi.1998.1111

