# Corrigendum

**DOI:** 10.1080/21501203.2018.1505371

**Published:** 2018-08-21

Awad NE, Kassem HA, Hamed MA, El-Feky AM, Elnaggar MAA, Mahmoud K, Ali MA. 2018. Isolation and characterization of the bioactive metabolites from the soil derived fungus *Trichoderma viride*. Mycology. https://doi.org/10.1080/21501203.2017.1423126

When the above article was first published online, one of the co-authors name was incorrectly spelt as Amal M. Elfiki instead of Amal M. El-Feky. This has now been corrected in both the print and online versions.

The authors apologize for this error.

